# Satellite Network Security Routing Technology Based on Deep Learning and Trust Management

**DOI:** 10.3390/s23208474

**Published:** 2023-10-15

**Authors:** Zhiguo Liu, Junlin Rong, Yingru Jiang, Luxi Zhang

**Affiliations:** Communication and Network Laboratory, Dalian University, Dalian 116622, China; rongjunlin@s.dlu.edu.cn (J.R.); jiangyingru198@163.com (Y.J.); 15242561513@163.com (L.Z.)

**Keywords:** trust management, D–S evidence theory, variational autoencoder, secure routing

## Abstract

The conventional trust model employed in satellite network security routing algorithms exhibits limited accuracy in detecting malicious nodes and lacks adaptability when confronted with unknown attacks. To address this challenge, this paper introduces a secure satellite network routing technology founded on deep learning and trust management. The approach embraces the concept of distributed trust management, resulting in all satellite nodes in this paper being equipped with trust management and anomaly detection modules for assessing the security of neighboring nodes. In a more detailed breakdown, this technology commences by preprocessing the communication behavior of satellite network nodes using D–S evidence theory, effectively mitigating interference factors encountered during the training of VAE modules. Following this preprocessing step, the trust vector, which has undergone prior processing, is input into the VAE module. Once the VAE module’s training is completed, the satellite network can assess safety factors by employing the safety module during the collection of trust evidence. Ultimately, these security factors can be integrated with the pheromone component within the ant colony algorithm to guide the ants in discovering pathways. Simulation results substantiate that the proposed satellite network secure routing algorithm effectively counters the impact of malicious nodes on data transmission within the network. When compared to the traditional trust management model of satellite network secure routing algorithms, the algorithm demonstrates enhancements in average end-to-end delay, packet loss rate, and throughput.

## 1. Introduction

Satellite networks possess characteristics such as open media, dynamic topology, and limited resources [1]. Consequently, the design of routing algorithms for satellite networks is currently a hot research topic, and numerous researchers have made significant contributions in this field. However, in terms of the security vulnerabilities inherent to routing protocols themselves, most existing satellite network routing protocols have not adequately addressed this issue. The existing efforts by researchers have primarily focused on ensuring the availability of satellite network routing. Current research on satellite network security mainly involves the transplantation of security techniques from terrestrial networks to counter threats. Although technologies such as intrusion detection, anomaly protocol detection, firewall techniques, and interference-eliminating channel coding [2,3,4,5] have a significant research background in terrestrial networks, the scarcity of resources at satellite nodes and the influence of the overall architecture of satellite networks mean that many techniques applicable to ground networks cannot be directly applied in satellite networks. Continuous research is necessary in the field of technological integration. After researchers thoroughly consider the characteristics of satellite networks and the applicability of security technologies, the field of satellite network security has gradually yielded numerous achievements. Among these, secure routing in satellite networks has received considerable attention.

The security of satellite network routing is primarily considered from two aspects: encryption and trust management [6]. Initially, researchers fortified routing data from a cryptographic perspective, which constitutes an external defense. Employing such defensive measures involves constant encryption and decryption of transmitted data by satellite nodes, leading to relatively low efficiency. Moreover, a critical flaw in this approach is its inability to prevent attackers from breaching encryption barriers and impersonating legitimate nodes. Once a satellite node is compromised as a malicious entity, encryption loses its protective effect on the satellite network, posing substantial threats to satellite network routing and jeopardizing the overall system’s security [7]. Therefore, in the current research on secure satellite network routing, countering internal attacks where attackers mimic normal nodes is a central concern. Traditional approaches to defending against internal attacks in satellite network routing involve trust management models, which can be simplified trust models using weighting and more complex mathematical trust models. With the emergence of various novel attack methods, the efficacy of traditional trust models in detecting malicious attacks has declined. This is due to their simplistic mathematical models that lack precision in modeling actual node attack behaviors. In the face of evolving attack methods, traditional trust models struggle to extract attack features effectively. Consequently, there is a pressing need to enhance traditional trust models. Furthermore, integrating machine learning into traditional trust models has been explored in the Internet of Things (IoT) [8]. However, using machine learning to improve models presents challenges such as obtaining datasets with attack labels, as many new attack types are unseen, making labeling difficult. Additionally, machine learning’s capability to extract data features is limited, particularly for deep-level attack methods. Deep learning, on the other hand, possesses high flexibility and adept data feature extraction capabilities, particularly in the domain of anomaly detection. Thus, this paper employs deep learning anomaly detection techniques [9] to reinforce and enhance traditional trust models, forming a novel intelligent security model. In addition, considering the focus on secure routing in satellite networks, the foundational routing algorithm requires enhancement. In this regard, the ant colony algorithm is chosen as the benchmark routing algorithm for satellite networks. This selection is due to the abundance of research applying the ant colony algorithm to satellite network routing, indicating its suitability within this context. Furthermore, as this paper intends to employ an intelligent security model to assess satellite node security, traditional static routing algorithms are unable to promptly integrate with the results of the intelligent security model. The ant colony algorithm, being an intelligent optimization algorithm, can dynamically carry the security assessment factor of the intelligent security model. Consequently, this paper combines the ant colony algorithm with the intelligent security model to jointly counter internal attacks in satellite networks while establishing a secure routing path. For the convenience of later elaboration, the proposed algorithm is named TVAE in this paper.

The innovation points of this article include the following three aspects:

(1) Due to issues such as electromagnetic interference, network congestion, and satellite node malfunctions, it is challenging to ascertain whether transmission failures are caused by malicious attacks in satellite networks. Addressing this challenge, this paper proposes the application of the Dempster–Shafer (D–S) evidence theory to handle uncertainties in satellite network communication data. By quantifying interaction data within the satellite network, direct trust vectors and indirect trust vectors are formed. The D–S evidence theory integrates these vectors to create a comprehensive trust vector, which mitigates the misjudgment of satellite network node behavior caused by nonmalicious factors.
(2) Traditional mathematical models, when used as security trust models, exhibit low detection accuracy and limited flexibility in identifying unknown attacks. Therefore, this paper introduces the variational autoencoder (VAE) from the field of deep learning to discern malicious node behavior. Furthermore, to enhance the VAE’s ability to detect anomalous behavior, an attention mechanism is incorporated into the VAE network. Specifically, after the D–S evidence theory processes communication data, the VAE network receives cleansed communication data as input to its encoder. By adjusting the weights of latent variables provided by the encoder using attention mechanisms, the VAE network can more accurately learn the underlying feature information of the trust vector, thus improving the detection of malicious node behavior and enhancing satellite network routing security.
(3) By incorporating the security scores derived from the VAE model into the pheromones of the ant colony algorithm, the satellite network can dynamically guide ants to circumvent malicious nodes during the routing process. Specifically, when ants choose the next hop, they consider their pheromones along with the security assessment factors of neighboring nodes. Ants are inclined to choose paths with higher products of pheromones and security factors. Thus, improving the ant colony algorithm with the security factor of the VAE model helps the satellite network establish secure routing paths.

The remainder of this paper is organized as follows. In the Section 2, the relevant research work on satellite network secure routing is introduced. In the Section 3, the structure of the secure intelligent model is analyzed and applied to the ant colony algorithm satellite network routing. In the Section 4, we carry out a simulation. The Section 5 is the summary and prospect of the article.

## 2. Related Research

D–S evidence theory has been widely discussed and used in recent years because it can combine uncertain information from different sources with different levels of abstraction. However, due to the problem of evidence conflict in this theory, many scholars have conducted relevant research on it. Reference [10] improved on the traditional D–S evidence theory by reassigning weight factors before evidence fusion to solve the problem of counterintuitive results when D–S evidence theory fuses highly conflicting information. Reference [11] proposed a new method for measuring global uncertainty, which not only retains the advantages of previous measurements but also has higher sensitivity and wider scope to changes in evidence. Reference [12] analyzed the relationship between D–S evidence theory and classical probability theory and then proposed a generalized evidence combination formula. The advantage of this method is that it can alleviate the requirement of evidence independence and make D–S evidence theory perform better in practical application. In addition, ref. [13] applied the D–S evidence theory to the sensor system of unmanned vehicles, so that unmanned vehicles could make better decisions in actual scenarios.

Since satellite nodes are exposed to an open space environment, satellite network routing protocols are inevitably subject to many attacks [14], which can be roughly divided into two types: although the encryption mechanism can effectively protect against external attacks, it cannot deal with the internal attacks of the satellite network. Therefore, a security protection method against internal attacks has been put forward by the people—trust management mechanism. With the development of malicious attack mode, the structure of the trust model has also risen from the simple weighted principle to the construction of various complex mathematical models. Reference [15] adopted a simple mathematical trust model, and its trust management model was based on the weighted average of trust values. In this paper, direct trust values, indirect trust values, and comprehensive trust values were established by using simple mathematical formulas for the interaction behaviors between satellite network nodes and neighbors, to determine the security of satellite network nodes. The advantage of this scheme is that it makes full use of the dynamic characteristics of the satellite and adopts adaptive adjustment strategies to adjust the trust value to detect malicious behaviors and respond quickly when the network fails. However, the trust evaluation model of this scheme uses the weighted average method to calculate the comprehensive trust value, so the evaluation of trust value is highly subjective and the evaluation results are not accurate. Reference [16] discussed the inference model based on the Bayesian network, which uses a set of current observations (i.e., direct experience) in Bayesian theory to predict the future state of the route, and its nodes estimate the parameters of the prior distribution through the collected recommendation information and combine the direct interaction to obtain a posterior distribution, based on which the trust value of each interval can be predicted. Since this scheme uses Bayesian theory to deduce the solution of trust value, its accuracy in the security assessment of nodes is greatly improved. However, the adoption of Bayesian theory requires the provision of prior probability and conditional probability, which may not be fully provided in the establishment of the security trust model. Reference [17] proposed a fuzzy trust model based on experience and rationality, which accesses the accuracy and integrity of messages through fuzzy logic. It uses ID authentication to evaluate whether a message is authorized and uses the relevant data stored in the nearest node to measure the accuracy of the message. This scheme adopts a fuzzy trust management model, which has the advantage that it can make a more accurate judgment on the evaluation of the node trust value, but its disadvantage is that the accuracy of the evaluation results begins to decline as the object scale expands. To solve the routing security of low-Earth-orbit satellites, ref. [18] proposed the secure routing algorithm of the LEO satellite network based on node trust (SLT) algorithm, which is based on the distributed trust evaluation model. It calculates the direct trust, indirect trust, and comprehensive trust values among satellite nodes through the D–S evidence theory, and then combines the comprehensive trust value with the basic routing algorithm Orbit Prediction Shortest Path First Routing for Resilient LEO Satellite Networks (OPSPF) to effectively reduce the influence of malicious nodes. It improves the security of satellite network routing, but its disadvantage is that its satellite network routing algorithm is static and difficult to deal with faults. In [19], a decentralized trust management scheme (DTMS) was designed to filter out malicious nodes in satellite networks. This scheme combines the amount of forwarded evidence and the energy consumption rate of nodes to form direct trust and then establishes a trust framework. The novelty of this scheme is that the energy factor of satellite nodes is considered. However, a drawback of this method is that the calculation process of trustworthiness recommendation is complex, consuming a significant amount of computational and time resources. In the paper [20], the proposed algorithm improves the AODV protocol of ground networks based on the characteristics of satellite networks. Additionally, it combines this protocol with a security trust model to effectively counteract malicious attacks in satellite networks. However, the drawback of this algorithm is that the trust evaluation model it employs uses a Bayesian estimation model. The prior probabilities in the Bayesian estimation model need to be predetermined, which could lead to difficulties in achieving an optimal detection level for the established security trust model. Reference [21] introduced the trust mechanism into the vehicle joint network. Since the vehicle network has the problem of inconsistent trust in different regions, this paper proposed the combination of active detection, blockchain technology, and trust model to improve the effectiveness of the vehicle network system in detecting malicious nodes significantly. The drawback of this algorithm is that it applies to the field of the Internet of Things. The trust model in [22] was used to improve the security of wireless sensor networks. Due to the limitation of computing resources and energy resources of wireless sensors, a trust model scheme based on the Pareto frontier optimal solution was proposed in this paper, so that the security of wireless sensors could be better guaranteed. The drawback of this algorithm is that it involves significant computational and energy resource consumption. In [23], the trust model was applied to underwater acoustic sensors. To identify underwater malicious nodes more accurately, deep reinforcement learning was adopted to detect malicious behaviors in this study. Experimental results showed that, due to the adoption of deep learning, this method could detect malicious attacks at a deep level, which greatly ensures the safety of underwater acoustic sensors. The downside of this algorithm is that utilizing deep reinforcement learning to construct underwater sensors requires a substantial amount of computational power and energy consumption. Reference [24] proposed a secure routing scheme that activated trust. The main innovation of this scheme is to avoid black holes by actively creating multiple detection paths, quickly detecting and obtaining node trust, so as to improve the security of data paths. The drawback of this algorithm is that constructing multiple secure paths is not only time-consuming but also consumes a significant amount of node storage resources. Reference [25] proposed a space information network secure routing protocol based on intrusion detection. To enhance the accuracy of attack detection, this technique employs multiple nodes to identify malicious behavior, thus establishing a collaborative intrusion detection system. Additionally, this approach introduces a trust management system, which, by integrating intrusion detection and trust management, enables more precise identification of malicious nodes, thereby enhancing the security of satellite node routing. The advantage of this technique lies in its use of proactive intrusion detection, which offers heightened sensitivity and accuracy in detecting malicious behavior. However, its drawback is that the application of collaborative intrusion detection technology consumes a significant amount of satellite resources. Reference [26] proposed that in satellite networks, the trust measurement mechanism and routing selection strategy work effectively when the network load is balanced and light. However, uneven network load distribution is an inherent characteristic of satellite networks. Once the load increases, nodes with higher trust levels may forward an increasing number of packets, leading to congestion in certain nodes and degrading network performance. To address this, the paper introduced load-balancing techniques to enhance the trust management system. This enabled the satellite network to consider both network load and security simultaneously. However, a drawback of this approach is that the trust management system employed a simple mathematical model, resulting in lower accuracy in identifying malicious nodes. Reference [27] proposed a trust management model based on generative adversarial network (GAN) in an underwater sensor environment. This approach combined artificial intelligence and trust management models to effectively detect malicious behavior. Additionally, the paper considered the energy consumption of underwater sensors, achieving a balance between energy and security during the routing process. The advantage of this method lies in its utilization of an intelligent security trust model, providing strong assurance for the routing security of underwater sensors; however, its drawback is the relatively high resource consumption.

The anomaly detection module in the field of deep learning has a very good effect on the detection of malicious nodes, so many scholars have researched it. Reference [28] used the combination of the generative adversarial network (GAN), VAE network, and long short-term memory (LSTM) model to solve the problem that it takes a long time to find the optimal mapping from real-time space to potential space in the anomaly detection stage. To improve the performance of security monitoring, ref. [29] proposed a two-stage algorithm (S^2^-VAE) stacked fully connected variational autoencoder network model, which obtained excellent detection results on four public datasets. To solve the problem of poor interpretability of anomaly detection methods in satellite remote sensing test data, ref. [30] proposed an anomaly detection framework using causal network and feature-attention-based long short-term memory (CN-FA-LSTM). The proposed method is more interpretable than other commonly used prediction models, and its general applicability was verified on two common datasets. It was proposed in [31] that errors in satellite remote sensing test data would lead to false anomalies. To solve this problem, the deviation divide mean over neighbors (DDMN) was used in this study to model multivariate time series data by using a long short-term memory network, effectively avoiding false positives.

It is necessary to study the development of the ant colony algorithm because the ant colony algorithm is used in the basic routing in this paper. In [32], an improved ant colony algorithm was used to optimize the dynamic path given the shortcomings of the dynamic path optimization method. The experimental results showed that the running time of the optimal path obtained by this algorithm was obvious. In [33], an ant colony optimization algorithm based on the small-window strategy was proposed to solve the routing and wavelength allocation problems in satellite optical networks. The results showed that, compared with Dijkstra’s algorithm, this algorithm improved the system resource utilization by 45%. To meet the quality of service (QOS) requirements of end-to-end delay, link utilization, and bandwidth, ref. [34] proposed a combination of heuristic algorithm and ant colony algorithm to provide a better QOS guarantee. Experiments showed that this algorithm can provide more QOS guarantees than the shortest path algorithm.

The subject of this paper is secure routing in satellite networks. To provide a clearer explanation, the bullet points of the methods proposed in the literature for secure routing are presented in the form of a table below.

In Table 1, Internet of Things represents algorithms applicable to the field of the Internet of Things. Satellite network represents algorithms applicable to the field of satellite networks. Traditional trust model indicates that the algorithm uses a trust model without employing artificial intelligence algorithms. Intelligent trust model signifies that the algorithm employs a trust model utilizing artificial intelligence algorithms. Energy factor signifies that the algorithm takes both security and energy consumption factors into account. Lastly, resource consumption signifies that the algorithm has a relatively higher consumption of computational resources. √ indicates that the reference in the row of √ matches the characteristics of the column in which it resides.

## 3. Problem Modeling

### 3.1. Satellite Network Model

**Figure 1:**
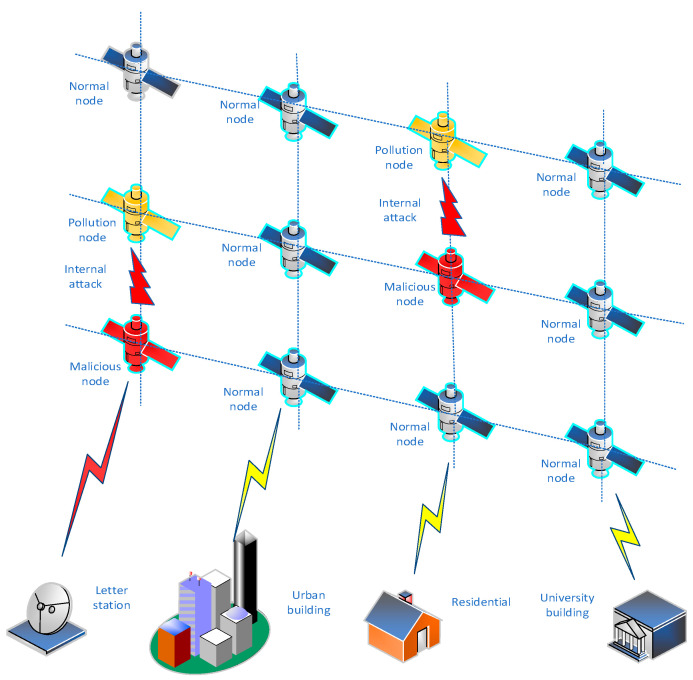
Satellite network routing attack architecture.

As shown in Figure 1, the satellite network routing attack architecture consists of the low-Earth-orbital satellite (LEO) network and the corresponding ground infrastructure (urban building and satellite receiving stations). Among them, the nodes of the LEO will be attacked by routing, which will pollute the data flow, and then affect the data communication of the entire space–Earth network. The red satellite node in the figure indicates that it has been invaded and become a malicious node. The yellow satellite node indicates a communication interruption or information loss due to a malicious attack by a red satellite node. The remaining blue satellite nodes indicate normal satellite nodes. Because the satellite network will be attacked by malicious nodes during the routing process, this article uses a trust model to strengthen the routing algorithm. In this way, the satellite network route established by the trust model can actively identify and avoid malicious nodes, and can effectively establish a secure route, thus improving the security of the satellite network route.

### 3.2. Security Trust Model

**Figure 2:**
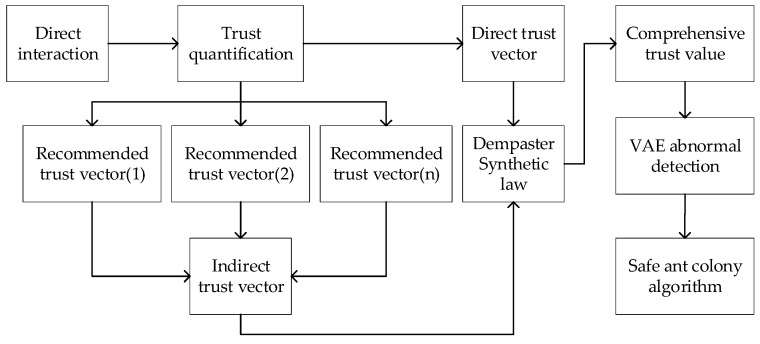
Satellite network security trust model.

Since the satellite network is subject to internal attacks during the routing process, to cope with such malicious attacks, this paper proposes to build a security trust model on the satellite to reinforce the routing. The structure is shown in Figure 2. To identify the malicious attack behavior, the interaction information between nodes should be collected and should be used as trustworthy evidence by the quantity of interaction information, then the direct trust and indirect trust vectors can be established. When the VAE network is trained, it needs a clean trust vector as a dataset, so this paper first uses D–S evidence theory to clean the trust vector. After the VAE network is trained successfully, the VAE network is used to identify the collected trust evidence for malicious purposes. Finally, the ant colony algorithm can use the security factor to combine with its pheromone in the routing process, so as to identify malicious nodes and establish a secure route.

### 3.3. Trust Evidence Data Cleaning

To assess the security of satellite network nodes, it is necessary to collect interaction information among the nodes. For internal attacks on the satellite network, the most commonly used credible evidence is the number of successful communication instances and the number of communication failures between nodes.

Due to the unique environment of satellite networks, it is not suitable to utilize a centralized trusted authority to compute the trust values of satellite nodes. Consequently, this paper employs a distributed collaborative approach among satellite nodes to calculate their trust values. When a satellite node evaluates the trustworthiness of its neighboring forwarding satellite nodes, it needs to track their historical packet forwarding behavior. Therefore, to track the historical forwarding behavior of the evaluated satellite node, this paper introduces a two-hop acknowledgment mechanism [18]. The mechanism operates as follows: If the evaluating node receives acknowledgment messages from both its neighbors and the neighbors of its neighbors within a specified timeframe, it indicates that the evaluated neighboring node has successfully forwarded the packet. If, within the specified timeframe, the evaluating node neither receives acknowledgment messages from its neighbors nor from the two-hop neighbors, it implies that the evaluated neighboring node explicitly refused to forward the packet. If the evaluating node within the specified timeframe only receives acknowledgment messages from its neighbors but not from the two-hop neighbors, it cannot determine whether the neighboring node successfully forwarded the packet. Therefore, based on the utilization of the two-hop acknowledgment mechanism, this paper initially defines the following formula:

(1) $$\;a=\frac{s}{s+f+u}$$

(2) $$b={\frac{f}{s+f+u}}_{}^{}$$

(3) $$c=\frac{u}{s+f+u}$$

where s represents the count of successful forwarding, f represents the count of failed forwarding, and u represents the count of cases where successful forwarding is uncertain. Additionally, a stands for the success forwarding rate, b stands for the failure forwarding rate, and c stands for uncertainty.

To alleviate the influence of satellite networks due to factors such as electromagnetic radiation and cybersecurity, the D–S evidence theory is used to correct the above trust evidence. Given a recognition framework Ω={T,∼T}, where T means trust, ∼T means dislike, m{T} represents the probability ratio of successful communication, m{∼T} represents the probability ratio of failed communication, and m{T,∼T} means the probability ratio of uncertain environmental factors, the calculations of the three can be calculated based on the above Formulas (1)–(3).

The definition of a direct trust vector is DR={m{T},m{∼T},m{T,∼T}. In the beginning, because the nodes in the satellite network have not had interaction with other nodes, the result of the DR here is {0,0,1}. The three components of the vector, respectively, represent the probability ratio of trust, the probability ratio of distrust, and the probability ratio of uncertainty. Here, 1 indicates that no communication evidence has been collected at the beginning, and it is impossible to determine whether the satellite node should be trusted.

The trust model of this article adopts a periodic update method, so the node update cycle can be set to Δt. If the current $DR_{ij}$ ($t_{n}$) passes after Δt, it will change to $DR_{ij}$ ($t_{n+1}$). $DR_{ij}$ represents the direct trust vector, and $t_{n}$ represents the current time point.

To enable the node to dynamically adjust the direct trust value of the neighbor node, the corresponding reward and punishment function can be set:

(4) $$DR_{ij}(t_{n+1})=(1-v)DR_{ij}(t_{n})+v\cdot DR_{ij}(t_{n+1})$$

The reward and penalty function uses parameter v to adjust the ratio between the past direct trust value and the next time point direct trust value. This allows satellite network nodes to dynamically assess the behavior of nodes and, consequently, evaluate whether the node’s behavior is secure. For the setting of two direct trust values, m{T} can be used to compare the difference with zero. If it is less than zero, a larger value will be assigned to v, so that the direct trust of the satellite node to the target node will soon slip down; if it is greater than zero, a smaller value will be assigned to v, so as to improve the direct trust value of the target node.

For the calculation of the recommendation trust vector $IR_{ij}$, we can use the direct trust vector of all neighbors of the evaluated target node. The recommended trust vector can be obtained by designing a special routing package to request the direct trust evaluation of the neighbor nodes.

Finally, comprehensive trust vectors synthesize the direct trust value and the recommended trust value through the following formula to obtain the comprehensive trust value.

(5) $$\left\{\begin{array}{l} m_{ij}^{*}(A)=((m_{n1,j}(A)⊕\ldots )⊕m_{nk.j}(A))⊕m_{ij}(A),A\in \Omega \\ m_{ij}^{*}(\emptyset )=0 \end{array}\right.$$

In Formula (5), the following two formulas are used for calculation when different evidence is synthesized:

(6) $$m_{1}(A)⊕m_{2}(A)=\frac{\sum_{X\cap Y=A}m_{1}(X)m_{2}(Y)}{1-K}$$

(7) $$K=\sum_{X\cap Y=\varphi }m_{1}(X)m_{2}(Y)$$

Finally, a comprehensive trust vector can be obtained through D–S evidence theory, and then its comprehensive trust value can be obtained. To obtain the dataset of training variational autoencoder networks, trust vectors need to be designed. The trust vector is formed by recording the comprehensive trust value with length $l_{w}$, and a large number of such trust vectors can be accumulated for the training of deep learning network models. Since the VAE-based anomaly detection model requires the input of normal data, a large number of trust vectors can be generated as normal datasets without malicious node interference at the initial stage of satellite network construction, and these data can be input into the variational autoencoder for model training.

### 3.4. Intelligent Security Trust Model

The variational autoencoder is an unsupervised model. Although its structure is similar to the self-coder, the principle is very different. The potential vector obtained by VAE is determined, and it cannot generate vectors that differ from the original input, so its generation process can be regarded as a discrete situation, while the encoder of variational autoencoder learns the distribution of input data, including mean and variance, and in this process, the noise of normal distribution can be added to enhance the uncertainty of the system. And because it learns the distribution of the data, the intermediate variables are in a continuous state, so there can be small changes when generating the output. Because of this, reconfiguration probability can be used to calculate outliers during anomaly detection, which is more conducive to the accuracy of results.

The principle of the variational autoencoder model framework with attention mechanism can be divided into the following processes: first, multiple intermediate continuous potential vectors are generated in the coding process; secondly, by introducing the attention mechanism to record the temporal relationships of different intermediate vectors, the VAE network can perform a weighted summation of intermediate latent variables to obtain the final latent variable. Among them, the vector that records the correlation between time is $q_{c}$. The weighted product of the $q_{c}$ vector and multiple intermediate vectors can obtain the potential vector with the time-important information. Then, the final potential vector is input into the decoder for training to obtain the final intelligent security trust model. In a word, the method extracts the time importance information of the original sample, and the detection accuracy is improved compared with the VAE network without the attention mechanism.

**Figure 3:**
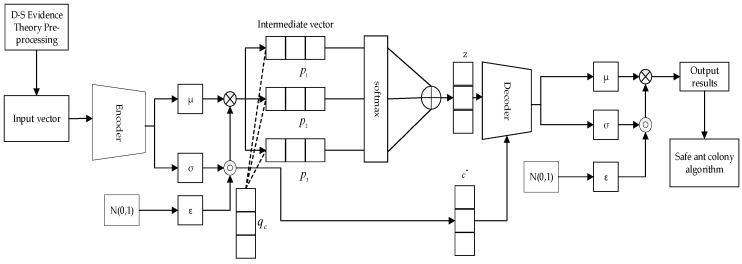
VAE abnormal detection model.

The trust vectors have been cleaned by D–S evidence theory to be $T_{c}=\{l_{1},l_{2},l_{3}\cdots l_{w}\}$, which has w dimensional, where the component of the trust vector represents the comprehensive trust value of the continuous different moments. Multiple historical trust vectors can be used in Figure 3 and fed to the encoder of the VAE module, which can reduce their dimensions to generate multiple compressed potential vectors that record the characteristic information of the original trust vector.

To capture the temporal relationship between trust vectors using the attention mechanism, the encoder can generate multiple intermediate potential variables after processing the trust evidence, that is:

(8) $$g(x_{i})=\{p_{1},p_{2},\cdots ,p_{n}\}$$

where $p_{j}(j=1,2,\cdots ,n)$ are the compressed vectors obtained by the encoder. Assume that the query in the attention mechanism follows the standard Gauss distribution, which can generate the final potential variable by extending the attention mechanism into an intermediate potential variable. This process is divided into three steps.

In the first step, we calculate the cosine similarity of $q_{c}$ and $p_{j}$:

(9) $$S_{j}=\frac{q_{c}\times p_{j}}{\left||q_{c}||\times ||p_{j}|\right|}$$

Due to the limited resources in satellite networks, this paper employs the 2-norm to calculate the values of $\left||q_{c}|\right|$ and $\left||p_{j}|\right|$ in Equation (9). The calculation formula is shown as follows:

(10) $$||y||={\left(|y_{1}{|}^{2}+\ldots +|y_{n}{|}^{2}\right)}^{\frac{1}{2}}$$

In the second step, the required weight of the attention mechanism can be calculated as follows:

(11) $$h_{j}=\frac{\exp\;(S_{j})}{\sum_{j=1}^{n}\exp(S_{j})}$$

In the third step, after obtaining and corresponding alignment rights, the final potential variables z can be obtained as follows:

(12) $$z=\sum_{j=1}^{n}h_{j}\times p_{j}$$

In the fourth step, the loss function is set as follows:

(13) $$L(\theta ,\varphi ;x)=E_{q_{\varphi }(z|x)}[\log p_{\theta }(x|z,c^{*})]-D_{KL}(q_{\varphi }(z,c^{*}|x)||p(z,c^{*}))$$

The training of the VAE model can be carried out by a backpropagation algorithm, and the reconstruction probability is generally calculated by the Monte Carlo technique and reparameterization method. According to other studies, VAE can be calculated by the average reconstruction error of multiple sampling times based on prior probability, which can be expressed as follows:

(14) $$m=\frac{1}{L}\sum_{i=1}^{L}p_{\theta }(x|\mu_{i}(x),\sigma_{i}(x))$$

where L represents the number of sampling times. A variable division self-encoder can obtain an average reconstruction error of m. The m can be directly transmitted to the ant colony algorithm to guide the ants to find the way.

### 3.5. Safe Ant Colony Algorithm

In the process of using the traditional ant colony algorithm to find the way, the satellite network does not consider security, and its initialization process is not efficient, so it is prone to stagnation. Therefore, the formula for selecting the next hop node can be modified to improve the efficiency of the ant colony algorithm.

First of all, when the satellite node starts to initialize the routing table, we do not forward the ant packet with random probability, because according to the predictability of the topology of the satellite network, the distance and direction of the current target satellite and the source satellite can be calculated in advance. The distance and direction between the target node and the source node can be calculated in advance to select the nearest neighbor node to forward the packet, which can reduce the initial pathfinding time of satellite network nodes and improve the overall efficiency of the system.

In the routing process of the ant colony algorithm, the formula of finding the next hop node is modified in this paper, so that the ant colony algorithm not only considers the influence of pheromone but also the security of the next hop node in the routing process. The following formula is improved:

(15) $$P_{ij}^{k}=\frac{\tau_{ij}{}^{\alpha }\cdot w_{ij}^{\beta }}{\sum_{j\in M}\tau_{ij}{}^{\alpha }\cdot w_{ij}^{\beta }}$$

(16) $$P_{ij}^{k}=\frac{{\left(\tau_{ij}\cdot m_{ij}\right)}^{\alpha }\cdot w_{ij}^{\beta }}{\sum_{j\in M}{\left(\tau_{ij}\cdot m_{ij}\right)}^{\alpha }\cdot w_{ij}^{\beta }}$$

where $P_{ij}^{k}$ represents the probability of the ant k moving from node i to node j, indicating that the current ant is at node i. M represents the set of nodes that the kth ant has not yet visited. $\tau_{ij}$ represents the concentration of pheromones on path ij. $w_{ij}$ represents the heuristic factor of the ant colony algorithm on path ij. α and β are two predefined parameters used to weigh pheromones and visibility, respectively. $w_{ij}$ is calculated as follows:

(17) $$w_{ij}=\frac{1}{d_{ij}}$$

where $d_{ij}$ indicates the distance from node i to j.

The original ant pathfinding Formula (15) only considers the pheromone concentration and the visibility between nodes in the pathfinding process, where the visibility between nodes can be recorded in the form of a matrix at the beginning. The revised Formula (16) combines the security assessment factor $m_{ij}$ stored in the node i with its pheromone. Specifically, this paper adopts a distributed trust management model, so the node i only records the security evaluation factors of neighboring nodes and does not obtain the security evaluation factors of other nodes across the neighbors. When the ant is pathfinding, the node where the ant is located has stored the security assessment factor of the neighboring nodes for a period of time, and the ant can comprehensively consider the probability of the next hop according to the pheromone concentration, security assessment factor, and visibility of node i and node j. If visibility is low or the product of the safety assessment factor and pheromone goes to zero, the ant abandons the path and tries to find a more efficient and safer path.

To avoid the ant colony algorithm falling into local optimal without exploring new routes, the random probability q is set to compare with the fixed threshold r. If this q > r, then we randomly read the security information of the next jump node. We choose a neighbor node; if q≤r, we still choose the way according to the above formula.

(18) $$p_{ij}^{k}=\left\{\begin{array}{l} \mathrm{Uniform}\;\mathrm{probability}\;if\;q>r\\ \frac{{\left(\tau_{ij}\cdot m_{ij}\right)}^{\alpha }\cdot w_{ij}^{\beta }}{\sum_{j\in M}{\left(\tau_{ij}\cdot m_{ij}\right)}^{\alpha }\cdot w_{ij}^{\beta }}\;if\;q\leq r \end{array}\right.$$

Moreover, when returning to the backward ant, security factors should also be considered and the formula should be updated to the backward ant:

(19) $$\tau_{ij}(t+1)=(1-\rho )*\tau_{ij}(t)+\sum_{k=1}^{n}\Delta \tau_{ij}^{k}(t)$$

Formula (20) is a security-enhanced version of Formula (19):

(20) $$\tau_{ij}(t+1)=(1-\rho )*\tau_{ij}(t)+\sum_{k=1}^{n}\Delta \tau_{ij}^{k}(t)*m_{ij}^{}$$

where ρ represents the evaporation factor of pheromones in the ant colony algorithm. The calculation formula of $\Delta \tau_{ij}$ is as follows:

(21) $$\Delta \tau_{ij}^{k}=\frac{Q}{L_{k}}$$

where Q represents the constant and $L_{k}$ represents the total distance traveled by the ant k.

In Formula (19), $\Delta \tau_{ij}^{k}(t)$ represents the pheromone concentration contributed by the ant i on the path ij. The formula updates pheromone concentrations without recording safety factor information obtained by other ants. The ants in Formula (16) take security into account when they find the way, but Formula (19) for updating the pheromone does not take security into account, which will lead to deviations when the ants update the pheromone. In order to correspond to the pheromone in Formula (16), Formula (20) combines the pheromone left by N ants on path ij with the safety assessment factor. The meaning of this formula is the product result of the pheromone and safety assessment factor left by n ants passing through this path, and the product result and the pheromone volatile factor ρ jointly determine the concentration of the updated pheromone.

In this way, when the ants were renewed to update the pheromone, the security factors were considered to make the results more reasonable.

In short, the idea of virtual topology is used to convert the dynamic topology of a satellite network into static topology with time slice changes. The advantage of this is to simplify the interference of many complex factors in the topology changes as much as possible so that the traditional ant colony algorithm can run in different time slice ranges. The operation period of the satellite group of the polar orbit constellation is T, and there are $N_{s}$ satellite nodes in the same orbit. According to the theorem in [35], when a satellite node enters the polar circle within a certain time and closes the link between different orbits, another satellite node must move out of the polar circle and open the link between its different orbits within this time range. Therefore, we can follow the conclusion above and divide the scope of the time slice into $T/N_{s}$, and the topology of the satellite network in a time slice can be regarded as unchanged.

### 3.6. Satellite Network Security Routing Algorithm

For clearer expression, in the table below (Algorithm 1), the symbol e refers to the iteration count of the ant colony, $e_{\max}$ represents the maximum value of the ant colony iteration count, N represents the number of ants, and M represents the set of nodes that the current ants have not yet traversed.
**Algorithm 1:** Satellite network security routing algorithmInput: Set the source node S and destination node D Output: a satellite network security routing1. The satellite network is initialized and the nodes collect communication data 2. Generating a Comprehensive Trust Vector SR Using D–S Evidence Theory 3. Use SR to generate $T_{c}=\{l_{1},l_{2},l_{3}\cdots l_{w}\}$ and form a dataset for training VAE 4. The satellite node uses the VAE network to collect the security evaluation factor m of the theoretical node. 5.   While e < $e_{\max}$ 6.         Set the tabu list to empty 7.          For i = 1 to N do 8.            While Ant i has not reached D 9.              If M is not null 10.                  Select the next hop j based on the Formula (17) 11.                  Add j to the tabu list 12.              End if 13.            Terminate this search 14.            update pheromones according to Formula (19) 15.            End while 16.            i = i + 1 17.          End for 18.          e = e + 1 19.   End while 20. Generate a secure route

## 4. Experimental Simulation and Analysis

This article uses NS2 simulation software(ns-allinone-2.35) to achieve simulation testing of Algorithm 1. The parameters of the satellite network are shown in Table 2:

The basic idea of simulation is to first use Python tools to build a VAE neural network for training. As the VAE model adopted in this paper is used to detect data security at the routing level of the satellite network, the traditional ground open dataset is not suitable for use here. Therefore, the dataset needs to be made manually during the training of the VAE model, that is, the routing evidence of the ant colony algorithm is collected and processed by D–S evidence theory, and the synthetic vector is obtained and then provided to the VAE model. After that, the training results are input into NS2, and then the safe ant colony algorithm is established to find the way, and various parameters in the satellite network routing process are obtained by adding malicious attacks. Finally, the experimental results are exported, and the data are read and plotted using Python.

For VAE, the learning rate is an important parameter in the VAE model; a too-high learning rate will lead to rapid loss, while a too-small learning rate will lead to slow convergence or overfitting of the model. For the setting of the learning rate, this paper tries to use 0.1, 0.01, 0.001, 0.0001, 0.00001, and other test values, and determines that the learning rate is 0.001 after the test. We set n in the attention mechanism to 3.

In this study, we employed NS2 simulation software to model attack scenarios among satellite nodes, and the relevant parameters of the satellite network were shown in Table 3. Specifically, we initially constructed a regular node topology within NS2 using code to simulate communication between normal nodes. To introduce malicious nodes among the normal ones, we integrated relevant Tcl code for malicious attacks into the code of normal nodes. These malicious codes are preinstalled with the NS2 software. When the nodes execute the ant colony algorithm, malicious nodes intentionally discard received data packets, thereby introducing the effects of malicious attacks and disrupting the normal operation of the network.

These malicious nodes can initiate different types of attacks. A black hole attack involves discarding data packets with a 100% probability upon receipt. A slander attack provides false trust recommendations while computing indirect trust values. Additionally, a selfish attack deliberately drops data packets at a certain rate to conserve energy.

In our paper, we assigned black hole attacks to 5 nodes, slander attacks to another 5 nodes, and selfish attacks to the remaining 6 nodes within a network of 16 nodes. These malicious nodes integrated into the normal network are then utilized to assess the reliability of the intelligent security trust model proposed in this study. To test the situation where the satellite network is in the lower route of the malicious attack environment, malicious nodes need to be added in the process of its routine, and different types of attack behavior will be launched by malicious nodes. NS2 simulation software itself has a malicious attack code, so the number of malicious nodes can be set in the satellite network node environment from 0 to 16. These malicious nodes can launch witch attacks, black holes attacks, defamation attacks, selfish attacks, and other attacks. Therefore, under different types of malicious attacks, the performance of different algorithms can be compared and analyzed under evaluation indexes such as average end-to-end delay, packet loss rate, and throughput.

### 4.1. Settings of Ant Colony Algorithm Parameters

In the ant colony algorithm, the selection of different parameters has an important impact on the performance of the algorithm.

**Figure 4:**
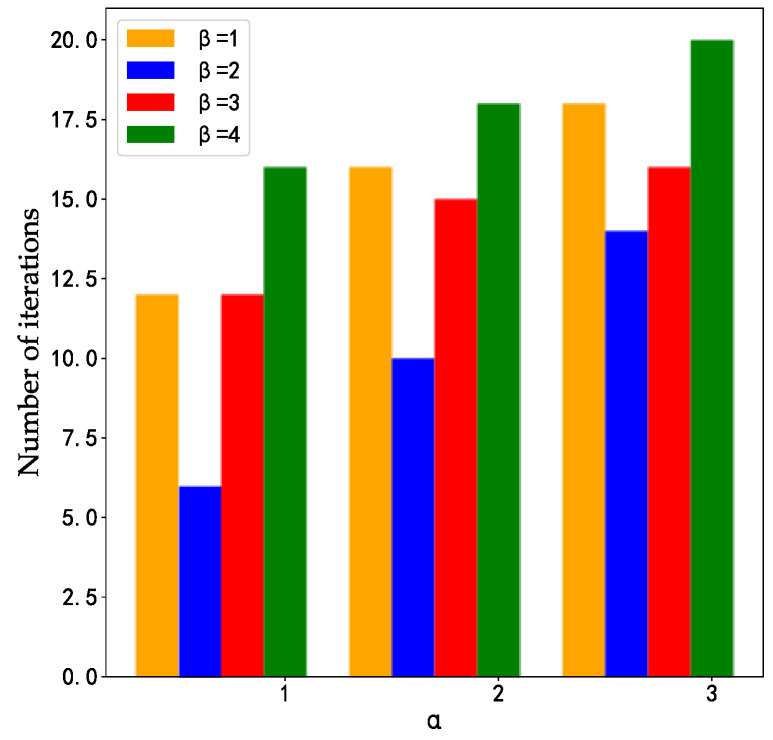
The values of pheromone α and heuristic factor β.

A larger information prime weight factor α will weaken the ability to search for other paths in the ant colony algorithm, while the smaller α value can easily cause the ant colony algorithm to fall into a locally optimal solution. Similarly, a larger inspiration factor β will weaken the role of pheromone in the algorithm process, leading to the local optimal solution; and the smaller β will turn the ant colony algorithm into a simple random search. Therefore, the appropriate value of α and β should be determined by experiments. According to the experimental results (Figure 4), we found that when α = 1, β = 2, the ant colony algorithm has the minimum number of iterations, so we chose this set of parameter values.

**Figure 5:**
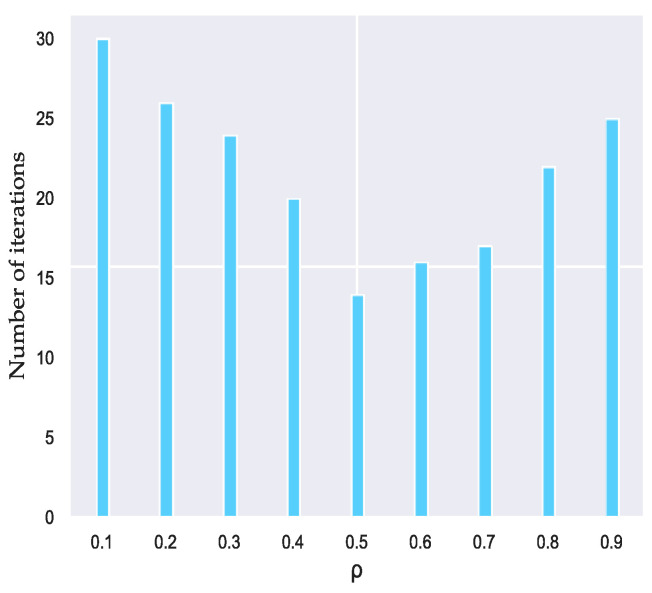
The value of volatile factor ρ.

In addition, the value of the volatilization coefficient of the pheromone ρ in Figure 5 directly affects the global search ability and convergence speed of the ant colony algorithm. The larger value will lead to the rapid volatilization of the searched path, increasing the probability of the ants choosing the duplicate path. The smaller ρ value will make the pheromone play slowly, thereby weakening the convergence of the algorithm. 

The value of the pheromone volatilization coefficient is determined by experiments. We find that when ρ = 0.5, the ant colony algorithm can achieve the optimal solution with the least iteration. Therefore, in the experiments of this article, the parameters of the ant colony algorithm are ρ = 0.5.

The settings of the ant colony algorithm’s hyperparameters, denoted as α and β, correspond to Formulas (18) and (20) mentioned earlier. The parameter α determines the weight of the product of pheromones and the security evaluation factor, while the parameter β determines the weight of the heuristic factor. The parameter ρ determines the magnitude of the pheromone evaporation factor. In the ant colony algorithm, when ants choose the next hop, they rely on Formula (18) to calculate probabilities. Ants are more inclined to search for paths with larger pheromone levels and higher security. Without the constraint of β, ants would disregard the distances between nodes during path selection. This could lead to most ants following certain fixed routes, potentially missing the optimal path. β, however, takes into account the distances between nodes. Consequently, ants have the opportunity to explore alternative paths during path selection, enabling the discovery of superior routes.

Furthermore, ρ primarily determines the update of ant pheromones. As indicated by Formula (20), the ant pheromones are not retained permanently on traversed paths; their evaporation is determined by the magnitude of ρ. Setting ρ too large or too small is unreasonable, as explained in the preceding text. Therefore, experimental data suggest that when α = 1, β = 2, and ρ = 0.5, the ant colony algorithm achieves optimal performance, ensuring the reliability of satellite network routing.

### 4.2. Analysis of Results

During the experiment, to simulate the malicious attack of the nodes in the satellite network, the route nodes can be randomly selected to become malicious nodes. The performance of the design scheme is tested by different performance indicators, and the average end-to-end delay, packet loss rate, and network throughput of the network are analyzed, respectively.

① Average end-to-end delay: The time taken from the source end node where the data packet starts to the destination node is calculated to completely accept the total time spent on the packet [18]:

(22) $$t_{delay}=t_{end}-t_{start}$$

② Package rate: The packet loss rate refers to the ratio of the number of packets lost in the process of data transmission to the number of packets sent [18]:

(23) $$t_{plr}=\frac{n_{loss}}{n_{send}}$$

③ Network throughput: Network throughput refers to the amount of node trans-mission data during the unit time without data packet loss [20]:

(24) $$th=\frac{S_{data}}{U_{t}}$$

#### 4.2.1. Average End-to-End Delay

To evaluate the efficacy of the algorithm utilized in this paper, it is designated as TVAE. Subsequent simulations will then compare the performance of TVAE with three other algorithms: ACO (Ant Colony Optimization) [32], TAODV (Trust Ad hoc on-Demand Distance Vector Routing) [20], and SLT (Secure Routing Algorithm of Leo Satellite Network-Based Node Trust) [18].

Average end-to-end delay refers to the time it takes for a packet to be sent from the source node to the destination node. After setting the number and type of malicious nodes in NS2 simulation software, the end-to-end delay data of satellite network routing can be obtained. As shown in the figure below, when there are no malicious nodes in the network, the benchmark algorithm ACO does not introduce a security trust model to harden it, thus saving additional computing time of trust value. Therefore, the average end-to-end delay of the ACO algorithm is the lowest among the four curves. TAODV, SLT, and TVAE all need to carry out security hardening of the trust model. Therefore, when there is no malicious node, the average end-to-end delay has a corresponding time consumption.

**Figure 6:**
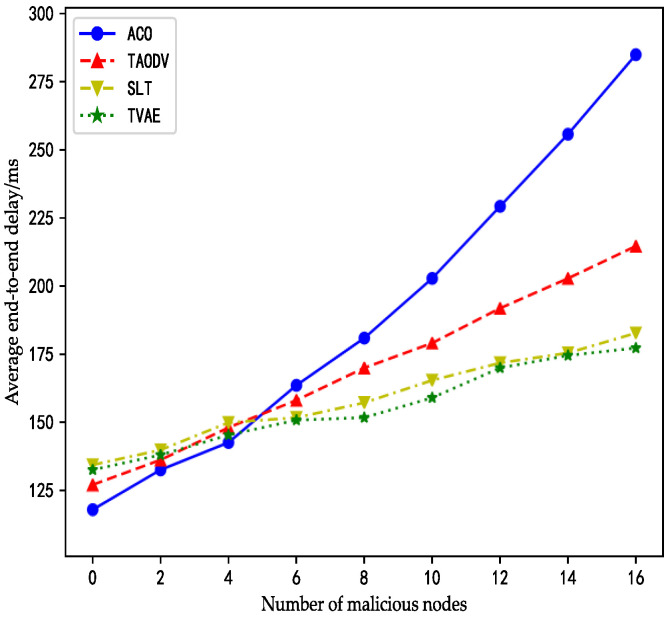
The impact of a malicious attack on delay.

In the beginning, because the number of malicious nodes is zero, the average end-to-end delay at this time is based on the execution time of the baseline routing algorithm and trust model. As evident from Figure 6, the average end-to-end delay of the ACO algorithm is 9 ms lower than that of the TAODV algorithm, 15 ms lower than the TVAE algorithm, and 18 ms lower than the SLT algorithm. The reasons for the trend of the four curves are as follows: Because the ACO algorithm does not have trust model reinforcement, it simply routes satellite network, so its average end-to-end delay is the smallest, while TAODV, because of the trust model adopted, is a traditional mathematical model which is relatively simple in trust calculation, so its average end-to-end delay is also small. In addition, when the SLT algorithm is running, in addition to running a basic routing module and dynamic trust evaluation processing module, it also uses a dynamic health diagnosis processing module to detect ISL faults, so its time consumption is larger than that of the TVAOD algorithm. The benchmarked algorithm of TVAE, the ant colony algorithm, needs to obtain the safety factors of the VAE module to guide its further routing in the routing process. However, TVAE does not consume the time of the dynamic health diagnosis processing module, so its final average end-to-end delay is slightly smaller than the SLT algorithm.

According to the illustration in Figure 6, as the number of malicious nodes increases to three, the curve of the TVAE algorithm crosses the curve of the TAODV algorithm. From this point onwards, when the number of malicious nodes exceeds three, the TAODV algorithm’s ability to counter malicious attacks becomes weaker than TVAE’s, consequently resulting in its average end-to-end delay surpassing that of TVAE.

Additionally, as the number of malicious nodes increases from zero to four, the ACO algorithm maintains a consistently low average end-to-end delay. The reason behind this outcome is that the ACO algorithm lacks a security trust model; therefore, it does not incur time overhead from security mechanisms. Moreover, due to the inherent capability of the ACO algorithm’s pheromones to dynamically perceive the quality of paths, it can dynamically choose alternative paths in the face of malicious attacks. Specifically, when a malicious node initiates a packet-dropping event, the ants in the ACO algorithm notice during multiple path exploration instances that this path is not viable, leading to minimal pheromone deposition on that path. Consequently, the ants abandon this path and prefer paths with higher pheromone levels. Ultimately, the other three algorithms need to account for the delay introduced by the security trust model. As a result, the ACO algorithm maintains a lower average end-to-end delay compared to the other three algorithms.

When the number of malicious nodes reaches five, the ACO algorithm’s self-regulation capability through pheromones is no longer competitive against the other three algorithms that possess security trust models. As depicted in Figure 6, it is evident that when the number of malicious nodes exceeds five, the curve of the ACO algorithm experiences a steep rise, indicating its inability to withstand malicious attacks.

As the number of malicious nodes in the satellite network increases, the ACO algorithm does not consider the security of routing. As can be seen from the following figure, the performance of the satellite network decreases sharply due to the attack behavior of malicious nodes, and the delay of data packets reaching the destination node increases significantly. This is because malicious nodes launch witch attacks, black hole attacks, forwarding attacks, and other attacks so that packets are maliciously intercepted and then transferred to the destination node, or malicious packet discarding causes the source node to retransmit packets, which will bring huge delay consumption. In contrast, because the other three algorithms adopt the security trust model to harden the routing process, they can cope with these malicious attacks better after malicious nodes launch attacks, so that the delay consumption is still within the acceptable range. Among them, TAODV and SLT, as the trust model adopted is the traditional mathematical model, have a large missing rate of malicious nodes. Therefore, as the number of malicious nodes increases, its effect is no longer as pronounced as in the TVAE used by neural networks. Therefore, TVAE has the lowest average end-to-end delay when dealing with malicious node attacks. Further observing the curve, it can be found that with the increase of malicious nodes, the growth rate of the SLT algorithm and TVAE does not change much, while the effect of TAODV is slightly worse. This is because the SLT algorithm consumes a certain amount of time in the calculation of trust value, but the benchmark algorithm of the SLT algorithm is OPSPF. It adopts a predictive way to calculate the routing table in advance, so it saves the calculation time of routing, and its speed is also very fast.

#### 4.2.2. Packet Loss Rate

The packet loss rate refers to the ratio of the number of packets lost in the process of data transmission to the number of packets sent. As can be seen from the figure below, when there are no malicious nodes in the satellite network, the packet loss rates of the ACO algorithm, TAODV algorithm, SLT algorithm, and TVAE algorithm are not much different. At this point, packet loss may occur due to normal factors such as network topology changes, or it may be caused by queue congestion.

As the proportion of malicious nodes in the network increases gradually, the packet loss rate of each algorithm also increases. Because the ACO algorithm does not consider security factors, it is unable to cope with malicious node attacks. Malicious nodes usually launch behaviors such as black hole attacks to discard packets, or for selfish attacks to discard certain specific packets. These attacks will cause the packet loss rate of the algorithm to rise sharply, seriously affecting the normal communication of data. Therefore, the packet loss rate of the ACO algorithm can be seen in the figure with a large increase. When the number of malicious nodes increases to 14, the packet loss rate exceeds 50%. At this time, the algorithm is already in a functional failure state, and the network built by it cannot carry out data communication.

However, the other three algorithms use the trust model to harden their routing algorithms, so they can avoid malicious nodes as much as possible in the routing process, to reduce the damage of malicious nodes to the satellite network. Therefore, the packet loss rate increases slightly when the number of malicious nodes increases. When four malicious nodes begin to appear in the network, the packet loss rate of the SLT algorithm and TVAE algorithm is almost 5%, the packet loss rate of the TAODV algorithm is 11%, and the packet loss rate of the ACO algorithm has reached 18%. Later, with the increase of malicious nodes, when malicious nodes reach 16, the packet loss rate of the TAODV algorithm is 28%, the packet loss rate of the SLT algorithm is 18%, and the packet loss rate of the TVAE algorithm is 12%.

The above results show that the TAODV algorithm, SLT algorithm, and TVAE algorithm all have a certain ability to resist malicious nodes in the network, so the packet loss rate of these three algorithms shows the same trend with the increase of the number of malicious nodes. However, since the TVAE algorithm adopts a novel neural network to detect malicious nodes, it can identify malicious nodes well and guide ant colonies to actively avoid these harmful nodes in the process of pathfinding in advance, so the possibility of being attacked is minimized. Therefore, its packet loss rate can be stabilized in a small range with the increase of malicious nodes.

**Figure 7:**
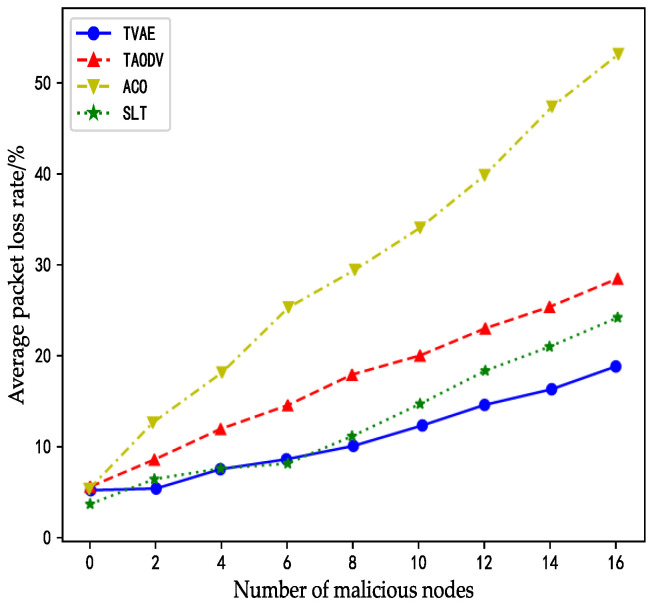
The impact of a malicious attack on the package rate.

Furthermore, as depicted in Figure 7, the four curves exhibit separation and no longer intersect as the number of malicious nodes increases. In terms of the overall trend, with a greater number of malicious nodes, the ACO algorithm’s curve experiences a larger upward incline, while the curves of the other three algorithms rise more gradually. The reasons behind these outcomes are as follows:

The ACO algorithm lacks a security trust model to reinforce the security of its routing, which positions its curve at the top of Figure 7. The TAODV algorithm, due to its utilization of a trust model belonging to traditional mathematical models, demonstrates limited effectiveness against malicious attacks and cannot match the capabilities of the SLT and TVAE algorithms.

Additionally, although the trust model of the SLT algorithm also employs traditional mathematical models, the algorithm itself is equipped with a fault detection mechanism, providing supplementary assistance to the trust model in combating malicious attacks. The TVAE algorithm adopts an intelligent security trust model, allowing it to effectively bypass malicious nodes when facing malicious attacks. Consequently, it maintains a lower packet loss rate, positioning its curve at the bottom of Figure 7.

#### 4.2.3. Network Throughput

The amount of network throughput refers to the maximum rate the system can accept without losing data packets. Bit/second or byte/second represent the throughput test results. In the following graphs, the number of malicious nodes affects the throughput of the network. As the number of malicious nodes increases, the average network throughput of the network exhibits a decreasing trend.

When the network was maliciously attacked, because the ACO algorithm did not adopt any security trust mechanism, it was difficult to deal with the attack behavior of malicious nodes. Therefore, the number of throughputs increased by the number of malicious nodes. It is equipped with a trust mechanism, so it can be identified in advance when dealing with malicious attacks to slow down the effect of malicious attacks. Therefore, the three algorithms decreased without the ACO algorithm when there was an increase of malicious nodes.

In the same way, because the TAODV algorithm’s trust model is relatively simple, it has a general effect when dealing with malicious attacks. Therefore, its throughput was compared to SLT and TVAE algorithms. The SLT algorithm and its trust model are more complicated than TAODV, and there is a dynamic health detection module. Therefore, its throughput decreased moderately. Finally, the TVAE algorithm is used for abnormal detection and its trust model is more sensitive when detecting malicious nodes’ attacks, so it can effectively avoid the malicious packet loss attack launched by malicious nodes. Thus, its throughput declines the slowest.

**Figure 8:**
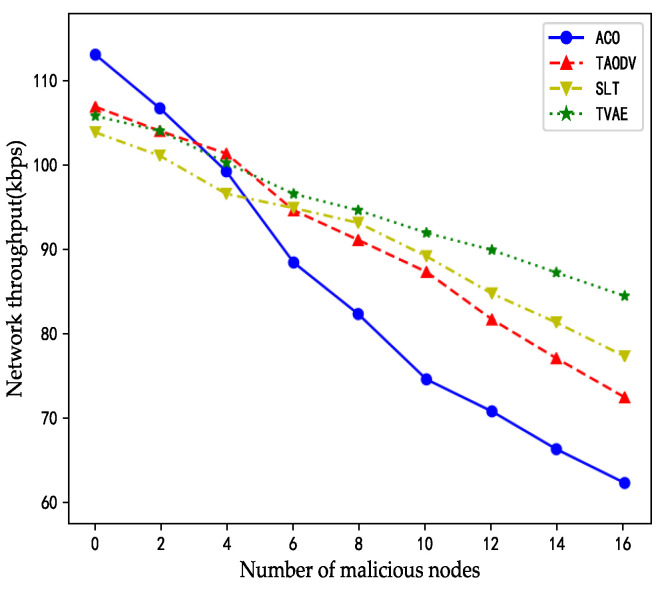
The effect of a malicious attack on throughput.

As indicated in Figure 8, when the number of malicious nodes is 0, the ACO algorithm exhibits the highest network throughput, surpassing the TAODV algorithm by 7 kbps, the TVAE algorithm by 8 kbps, and the SLT algorithm by 11 kbps. The reason behind this outcome is the absence of time overhead due to a security trust model in the ACO algorithm, allowing it to process more task requests within a unit of time. However, as the number of malicious nodes reaches four, the network throughput of the ACO algorithm becomes lower than that of the TAODV and TVAE algorithms. Furthermore, with an increase in the number of malicious nodes, the ACO curve steadily declines. Ultimately, when the number of malicious nodes reaches 16, the network throughput of the ACO algorithm drops to only 60 kbps.

This outcome is attributed to the fact that in the presence of packet loss caused by malicious attacks, the ACO algorithm’s probability of timeout and retransmission significantly increases. As a result, the quantity of tasks processed per unit of time by the ACO algorithm experiences a substantial decrease.

### 4.3. Analysis of Transmission Delay under Data Pressure

To test the latency of data transmission under different traffic conditions, this study increases the length of the packets themselves to provide a performance comparison of various algorithms in this scenario. Since the comparative algorithms require the running of a secure trust model, this paper selects a malicious node count of eight for processing here. Ensuring the presence of malicious interference makes the simulation results more convincing.

From the graph, it can be observed that as the packet size increases, the transmission latency of each algorithm also increases. Moreover, when the packet size reaches 800 B, the ACO algorithm’s transmission latency reaches 1560 ms, indicating that it consumes significant time in countering malicious attacks and may not be suitable for satellite network transmission. On the other hand, the other three algorithms, despite experiencing an increase in transmission latency, can maintain their latency stable at around 800 ms due to their security mechanisms in defending against malicious attacks. This ensures reliable data transmission even as data packet sizes increase.

### 4.4. Model Complexity Analysis

Time complexity analysis: To analyze the time complexity of the algorithm proposed in this paper, we break down the algorithm’s operational steps and gradually analyze their time complexities. As the D–S evidence theory’s time consumption in handling communication data is fixed, its time complexity is O(1). The VAE network’s time consumption in processing trust evidence data is also fixed, resulting in an O(1) time complexity during processing. In the context of the ant colony algorithm, let us discuss specific results based on the parameters established earlier in this paper. From the previous context, the number of satellite network nodes is $N_{s}$, the number of ants is N, and the number of iterations for the ant colony is $e_{\max}$. Considering the worst-case scenario, given the number of $N_{s}$ satellite network nodes, the lower limit for ants to find the destination node is NP steps. Meanwhile, when ants choose the next hop during their journey, there are $N_{s}-1$ potential scenarios. This implies that the worst-case scenario involves $N_{s}*(N_{s}-1)$ operations for a single ant’s pathfinding, resulting in a time complexity of $O(N_{s}^{2})$. Considering that the ant colony comprises N distinct ants, the time complexity for N ants’ pathfinding is $O(N_{s}^{2}*N)$. Moreover, since a single iteration through the ant colony’s pathfinding process might not yield the optimal solution, continuous iterations are necessary to achieve the optimal solution. With $e_{\max}$ iterations, the overall time complexity of the ant colony algorithm becomes $O(e_{\max}*N_{s}^{2}*N)$.

The above is an analysis of the time complexity of the algorithm proposed in this paper. Additionally, the additional time overhead introduced by the algorithm (TVAE) during practical operation is not significant and falls well within an acceptable timeframe. The specific reasons are as follows: The algorithm (TVAE) proposed in this paper operates in two distinct phases. The first phase involves satellite nodes assessing the security of their neighboring nodes. In the second phase, the satellite network executes the secure ant colony optimization algorithm. These two phases are executed separately, meaning that satellite nodes periodically evaluate the security of their neighboring nodes and store the security assessment factor m locally. When the secure ant colony algorithm is engaged in pathfinding, it solely requires the prestored local security assessment factor m. Furthermore, this action of accessing the security assessment factor m is executed with exceptional speed, resulting in minimal time consumption during the secure ant colony algorithm’s operation. Moreover, as depicted in Figure 6, when the number of malicious nodes is zero, the TVAE algorithm exhibits slightly higher average end-to-end latency compared to the ACO algorithm but lower than the SLT algorithm. Consequently, its runtime remains within acceptable limits.

Further, according to [32], the time complexity of the ACO algorithm is also $O(e_{\max}*N_{s}^{2}*N)$, which is the same as the time complexity of the TVAE algorithm proposed in this paper. However, because the TVAE algorithm introduces additional time overhead for looking up the security assessment factor m and combining it with pheromones compared to the ACO algorithm, the TVAE algorithm consumes slightly more time than the ACO algorithm. According to [20], the time complexity of TAODV is $O(N_{s}^{2}+X)$. Here, $N_{s}$ represents the number of satellite nodes, and X denotes the additional time overhead involved in trust calculations. The time complexity of this algorithm is lower than that of the TVAE algorithm, so the average end-to-end delay during the operation of the TVAE algorithm is slightly higher than that of TAODV. Finally, based on [18], the time complexity of the SLT algorithm is $O(N_{s}^{2}*M)+O(N_{s}*M)$, which is equivalent to $O(N_{s}^{2}*M)$. Its time complexity is similar to that of the TVAE algorithm, but due to the addition of the fault detection time overhead denoted by $O(N_{s}*M)$, its runtime is slightly slower compared to the TVAE algorithm.

**Figure 9:**
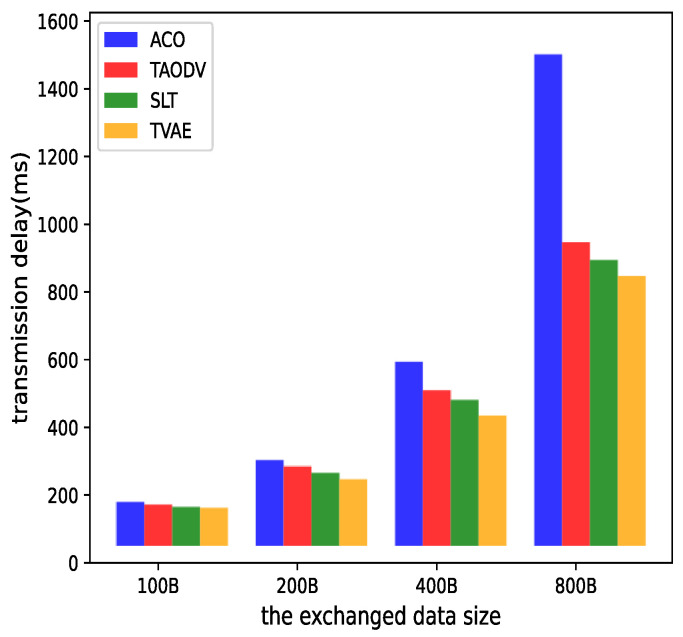
The effect of transmission delay in increasing the data packet.

As shown in Figure 9, when increasing the length of data packets and in the presence of malicious attacks, the TVAE algorithm can still maintain itself within an acceptable range. Therefore, the time consumption brought about by its security assessment module is not significant.

## 5. Conclusions

This article represents an advancement in the conventional trust model widely utilized in satellite network security routing. Given the open nature of satellite networks and their vulnerability to electromagnetic radiation interference, this paper takes a twofold approach. Firstly, it employs D–S evidence theory to mitigate these interference factors, thereby enhancing the accuracy of the trust vector. Secondly, it leverages the variational autoencoder architecture for identifying malicious behavior. Subsequently, the discriminant results are integrated into the ant colony algorithm to guide satellite network routing.

Currently, research at the intersection of satellite network security routing and deep learning remains relatively limited, and this paper presents pioneering efforts in this direction. In the future, further enhancements can be made to optimize the speed of satellite network security detection, thereby reducing time consumption during satellite network routing. Additionally, introducing the energy utilization factor of satellite network nodes into the network’s security model can strike a balance between energy efficiency and security detection. This can ultimately lead to an improved security routing strategy for satellite networks.

## Figures and Tables

**Table 1:** Comparison of secure routing.

Literature	Internet of Things	Satellite Network	Traditional Trust Model	Intelligent Trust Model	Energy Factor	Resource Consumption
[14]		√	√			
[15]	√		√			
[16]		√	√			
[17]		√	√			
[18]		√	√		√	√
[19]	√		√			
[20]		√	√			
[21]	√		√			√
[22]	√		√		√	√
[23]	√			√		√
[24]	√		√			√
[25]		√	√			√
[26]		√				
[27]		√		√		√

**Table 2:** Experimental simulation parameters.

Parameter Name	Parameter Value
Orbital altitude (km)	780
Number of orbital planes	6
Number of satellites in orbit	11
Orbital inclination (°)	86.4
Orbital plane spacing (°)	31.6
Interstellar link latitude threshold (°)	60
Number of interplanetary links per satellite	4

**Table 3:** Experimental simulation parameters.

Parameter Name	Parameter Value
Total number of satellite nodes	66
Number of malicious satellite nodes	16
Data packet length	100 B
Ant colony population size	100
Number of ant colony iterations	500
Trust vector length passed to VAE network $l_{w}$	10
Direct trust vector adjustment parameter v	0.5
Fixed threshold r	0.8
Trust evaluation model execution cycle(s)	2

## Data Availability

The processed data required to reproduce these findings cannot be shared as the data also form part of an ongoing study.

## References

[1] Cao X., Li Y., Xiong X., Wang J. (2022). Dynamic routings in satellite networks: An overview. Sensors.

[2] Van Nguyen T. (2012). Design of Capacity-Approaching Protograph-Based LDPC Coding Systems.

[3] Ma H., Fang Y., Chen P., Li Y. (2022). Reconfigurable Intelligent Surface-aided *M*-ary FM-DCSK System: A New Design for Noncoherent Chaos-based Communication. IEEE Trans. Veh. Technol..

[4] Shao S., Hailes P., Wang T.Y., Wu J.-Y., Maunder R.G., Al-Hashimi B.M., Hanzo L. (2019). Survey of turbo, LDPC, and polar decoder ASIC implementations. IEEE Commun. Surv. Tutor..

[5] Chen P., Shi L., Fang Y., Lau F.C.M., Cheng J. (2023). Rate-diverse multiple access over Gaussian channels. IEEE Trans. Wirel. Commun..

[6] Yan Y., Han G., Xu H. (2019). A survey on secure routing protocols for satellite network. J. Netw. Comput. Appl..

[7] Hao X.W., Ma J.F., Ren F., Liu X.Y., Zhong Y.T. (2011). A kind of authentication routing protocol based on double satellite network in space information network. Comput. Sci..

[8] Han G., He Y., Jiang J., Wang N., Guizani M., Ansere J.A. (2019). A Synergetic Trust Model Based on SVM in Underwater Acoustic Sensor Networks. IEEE Trans. Veh. Technol..

[9] Chalapathy R., Chawla S. (2019). Deep learning for anomaly detection: A survey. arXiv.

[10] Sun R., Huang H.Z., Miao Q. (2008). Improved information fusion approach based on DS evidence theory. J. Mech. Sci. Technol..

[11] Li R., Chen Z., Li H. (2022). A new distance-based total uncertainty measure in Dempster-Shafer evidence theory. Appl. Intell..

[12] Wu Y.G., Yang J.Y., Liu L.J. (1996). On the evidence inference theory. Inf. Sci..

[13] Qiao J., Zhang J., Wang Y. (2020). An improved multi-sensor D–S rule for conflict reassignment of failure rate of set. Soft Comput..

[14] Jiang C., Wang X., Wang J. (2015). Security in space information networks. IEEE Commun. Mag..

[15] Zhe L., Jun L. (2006). Research on secure routing of satellite network. J. Commun..

[16] Gao Y., Liu W. (2014). BeTrust: A dynamic trust model based on bayesian inference and tsallis entropy for medical sensor networks. J. Sens..

[17] Xu H., Zhang W., Li M. (2019). Secure Routing Scheme for Satellite Networks Based on Trust Management. IEEE Trans. Aerosp. Electron. Syst..

[18] Li H., Shi D., Wang W. (2022). Secure routing for LEO satellite network survivability. Comput. Netw..

[19] Asuquo P., Cruickshank H., Ogah C.P.A. (2018). A distributed trust management scheme for data forwarding in satellite DTN emergency communications. IEEE J. Sel. Areas Commun..

[20] Cai R.Y., Ju M.Y., Yang L., Pan C.S. Research on Lightweight Secure Routing Technology based on Satellite Network. Proceedings of the IEEE 2020 5th International Conference on Information Science, Computer Technology and Transportation (ISCTT).

[21] Li F., Guo Z., Zhang C. (2021). ATM: An active-detection trust mechanism for VANETs based on blockchain. IEEE Trans. Veh. Technol..

[22] Saad M.A., Jaafar R., Chellappan K. (2023). Variable-Length Multiobjective Social Class Optimization for Trust-Aware Data Gathering in Wireless Sensor Networks. Sensors.

[23] He Y., Han G., Jiang J. (2020). A Trust Update Mechanism Based on Reinforcement Learning in Underwater Acoustic Sensor Networks. IEEE Trans. Mob. Comput..

[24] Liu Y., Dong M., Ota K. (2016). ActiveTrust: Secure and trustable routing in wireless sensor networks. IEEE Trans. Inf. Forensics Secur..

[25] Cao S., Dang S., Zhang Y., Wang W., Cheng N. (2021). A blockchain-based access control and intrusion detection framework for satellite communication systems. Comput. Commun..

[26] Pan Y.H., Wang T., Wu Y., Wang W.H. (2011). Route Security Mechanism Based on Trust for Low Earth Orbit Satellite Network. Comput. Eng..

[27] Yang L., Yang S.X., Li Y., Lu Y., Guo T. (2022). Generative Adversarial Learning for Trusted and Secure Clustering in Industrial Wireless Sensor Networks. IEEE Trans. Ind. Electron..

[28] Niu Z., Yu K., Wu X. (2020). LSTM-based VAE-GAN for time-series anomaly detection. Sensors.

[29] Wang T., Qiao M., Lin Z. (2018). Generative neural networks for anomaly detection in crowded scenes. IEEE Trans. Inf. Forensics Secur..

[30] Zeng Z., Jin G., Xu C. (2022). Satellite telemetry data anomaly detection using causal network and feature-attention-based lstm. IEEE Trans. Instrum. Meas..

[31] Wang Y., Gong J., Zhang J. (2022). A deep learning anomaly detection framework for satellite telemetry with fake anomalies. Int. J. Aerosp. Eng..

[32] Deng X., Zeng S., Chang L., Wang Y., Wu X., Liang J., Ou J., Fan C. (2022). An ant colony optimization-based routing algorithm for load balancing in Leo satellite networks. Wirel. Commun. Mob. Comput..

[33] Cheng J. (2023). Dynamic Path Optimization Based on Improved Ant Colony Algorithm. J. Adv. Transp..

[34] Dong Y., Zhao S., dan Ran H., Li Y., Zhu Z. (2015). Routing and wavelength assignment in a satellite optical network based on ant colony optimization with the small window strategy. J. Opt. Commun. Netw..

[35] Werner M. (1997). A dynamic routing concept for ATM-based satellite personal communication networks. IEEE J. Sel. Areas Commun..

